# Hyponatremia Predicts New-Onset Cardiovascular Events in Peritoneal Dialysis Patients

**DOI:** 10.1371/journal.pone.0129480

**Published:** 2015-06-08

**Authors:** Hyung Woo Kim, Geun Woo Ryu, Cheol Ho Park, Ea Wha Kang, Jung Tak Park, Seung Hyeok Han, Tae-Hyun Yoo, Sug Kyun Shin, Shin-Wook Kang, Kyu Hun Choi, Dae Suk Han, Tae Ik Chang

**Affiliations:** 1 Department of Internal Medicine, Yonsei University College of Medicine, Seoul, Republic of Korea; 2 Brain Korea 21 for Medical Science, Severance Biomedical Science Institute, Yonsei University, Seoul, Republic of Korea; 3 Department of Internal Medicine, NHIS Medical Center, Ilsan Hospital, Goyangshi, Gyeonggi-do, Republic of Korea; Hospital Universitario de La Princesa, SPAIN

## Abstract

**Background and Aim:**

Cardiovascular (CV) disease is the leading cause of morbidity and mortality in patients on peritoneal dialysis (PD). Hyponatremia was recently shown to be a modifiable factor that is strongly associated with increased mortality in PD patients. However, the clinical impact of hyponatremia on CV outcomes in these patients is unclear.

**Methods:**

To determine whether a low serum sodium level predicts the development of CV disease, we carried out a prospective observational study of 441 incident patients who started PD between January 2000 and December 2005. Time-averaged serum sodium (TA-Na) levels were determined to investigate the ability of hyponatremia to predict newly developed CV events in these patients.

**Results:**

During a mean follow-up of 43.2 months, 106 (24.0%) patients developed new CV events. The cumulative incidence of new-onset CV events after the initiation of PD was significantly higher in patients with TA-Na levels ≤ 138 mEq/L than in those with a TA-Na > 138 mEq/L. After adjustment for multiple potentially confounding covariates, an increase in TA-Na level was found to be associated with a significantly lower risk of CV events (subdistribution hazard ratio per 1 mEq/L increase, 0.90; 95% confidence interval, 0.83–0.96; *p* = 0.003). Patients with a TA-Na ≤ 138 mEq/L had a 2.31-fold higher risk of suffering a CV event.

**Conclusions:**

These results provide evidence of a clear association between low serum sodium and new-onset CV events after dialysis initiation in PD patients. Whether the correction of hyponatremia for this indication provides additional protection for the development of CV disease in these patients remains to be addressed in interventional studies.

## Introduction

Hyponatremia is the most common electrolyte disorder and is an important marker of disease severity and poor prognosis in several medical conditions including advanced heart failure, liver cirrhosis, and chronic kidney disease (CKD) [1–10]. It is not clear whether hyponatremia per se increases the risk of adverse outcomes or is simply a surrogate marker of disease severity; however, several studies have shown a strong association between hyponatremia and high mortality in patients on maintenance hemodialysis (HD) [6–9]. In a recent study [10], we showed that hyponatremia was common in patients treated with peritoneal dialysis (PD) despite continuous dialysis therapy, and that low serum sodium concentration was an independent predictor of all-cause and infection-related mortality in such patients. Of note, cardiovascular (CV) disease is a significant risk and is among the leading causes of hospitalization and death in patients with end-stage renal disease (ESRD) on dialysis. Although CV disease is frequently present in patients commencing dialysis, the condition may develop during long-term treatment [11]. Thus, the identification of novel risk factors is critical for the prevention of CV disease in patients undergoing dialysis. In our previous study [10], patients with time-averaged serum sodium (TA-Na) level < 137 mEq/L showed a significantly higher cumulative incidence of CV death compared to those with a TA-Na ≥ 137; however, there was no significant relationship between TA-Na level and CV death in the multivariable analysis [10]. This weak statistical effect found in the previous study indicates a need for further in-depth investigation to establish the effects of low serum sodium on the CV risk with a different specific outcome such as CV events. Therefore, the present study examined the relationship between low serum sodium concentration and the development of CV events in the same cohort of PD patients used in our previous study [10].

## Methods

### Ethics statement

The study was carried out in accordance with the Declaration of Helsinki and approved by the Institutional Review Board of Ilsan Hospital Clinical Trial Center. We obtained informed written consent from all participants involved in our study.

### Patients and data collection

As described in detail previously [10], the study included 549 patients who commenced PD at Yonsei University Severance Hospital or NHIS Ilsan Hospital between January 2000 and December 2005. Patients < 18 years of age at the time of initiation of PD, or with < 6 months of follow-up, or who had been on HD or received a kidney transplant before initiation of PD, were excluded. Demographic and clinical data were collected at the commencement of PD. Laboratory and dialysis-specific data obtained at the time of dialysis adequacy assessment were considered to be baseline values and included serum sodium, potassium, and bicarbonate concentrations, hemoglobin, calcium, phosphorus, serum albumin, ferritin, serum C-reactive protein (CRP) levels; Kt/V urea, percentage lean body mass (%LBM), normalized protein catabolic rate (nPCR); PD ultrafiltration data; and residual glomerular filtration rate (GFR). All parameters were recorded longitudinally throughout the follow-up period and were calculated as averages of the means of measurements taken every 3 months for laboratory data, and every 6 months for dialysis adequacy parameters. Serum sodium concentration was measured using an electrode-based method (UniCel DXC 800; Beckman Coulter, CA, USA) and was corrected for serum glucose level using the following formula: Corrected sodium = measured sodium + 0.016 × (serum glucose—100) [12].

### Study outcomes

The study participants were followed-up to December 31, 2011. The primary outcome parameter was the composite of the first occurrence of a fatal or nonfatal CV event, defined as coronary artery disease (angioplasty, coronary artery bypass grafting, myocardial infarction, or angina); congestive heart failure; cerebrovascular disease; and peripheral artery disease (revascularization or amputation). The nature of CV event was established based on the onset of typical symptoms and signs, not based on the screening test. Congestive heart failure was defined as clinically typical symptoms and signs, such as newly developed dyspnea, shortness of breath, or raised jugular venous pressure combined with systolic or diastolic dysfunction by echocardiography. Cerebrovascular disease was defined as cerebrovascular infarction or hemorrhage confirmed both by typical clinical symptoms and imaging study; computed tomography or magnetic resonance imaging.

### Statistical analysis

The results are expressed as means ± standard deviations or as percentages. Data were analyzed using Student’s *t*-test and the χ^2^ test; ANOVA was used for multiple comparisons. The Kolmogorov-Smirnov test was used to determine the normality of the distribution of parameters. If data were not normally distributed, they were expressed as the median and interquartile range (or after log-transformation) and were compared using the Mann–Whitney test or Kruskal-Wallis test. Time to the first occurrence of a CV event after PD initiation was estimated and compared between groups based on the median (138.1 mEq/L) and tertiles (< 137, 137–139, and ≥ 139 mEq/L) of TA-Na levels using the cumulative incidence competing risk method and the K-sample test developed by Gray [13]. Data on switching to HD, kidney transplantation, loss to follow-up, and non-CV death were censored in analysis. To determine risk factors for the occurrence of new CV events, competing risks multivariate Cox’s regression was performed, as described by Fine and Gray [14]. In all survival analyses, the Cox’s model proportionality assumption was confirmed by testing of Schoenfeld residuals. The results are expressed as a subdistribution hazard ratio (SHR) with 95% confidence intervals (CIs). A *p*-value less than 0.05 was considered to indicate statistical significance. All statistical tests were conducted using the Statistical Package for the Social Sciences, version 21.0 (SPSS Inc., Chicago, IL, USA), and the R version 3.0.2 software package (http://cran.r-project.org/) including cmprsk library.

## Results

### Patient characteristics

Of the initial group of 549 patients, 108 were excluded, such that 441 patients were finally included in the study. Excluded patients were significantly older than and generally had higher comorbidity scores than the patients who were finally included. Other baseline characteristics did not differ between included and excluded patients. The demographic and clinical characteristics of the 441 study participants were detailed in previous our report Table 1 [10]. The patients who suffered from at least one among the four CV events before PD initiation were 123 (27.9%). Table 1 summarizes patient characteristics of previous CV disease and echocardiographic findings of parameters associated with hydration at initiation of PD according to TA-Na level tertiles, and showed no significant association between the type of previous CV disease or echocardiographic parameters and the TA-Na level.

**Table 1:** Patient characteristics at baseline.

	Time-averaged serum sodium (mEq/L)			
	< 137	137 to < 139	≥139	*p* for trend
Previous cardiovascular disease (n = 441)	48 (33.1)	41 (27.7)	134(23.0)	0.054
Coronary artery disease	27 (18.6)	24 (16.2)	19 (12.8)	0.176
Congestive heart failure	15 (10.3)	12 (8.1)	8 (5.4)	0.118
Cerebrovascular disease	24 (16.6)	25 (16.9)	20 (13.5)	0.473
Peripheral artey disease	9 (6.2)	1 (0.7)	5 (3.4)	0.187
Echocardiographic findings (n = 98)				
LA volume index (mL/m^2^)	32.1±11.1	30.4±17.6	27.8±8.9	0.177
LV end-diastolic diameter (mm)	50.0±7.4	49.3±4.8	51.9±5.4	0.137
LV mass index (g/m2)	118.8±36.2	117.6±36.9	120.0±36.9	0.884
TR peak velocity (m/sec)	2.32±0.42	2.29±0.37	2.31±0.36	0.916

Values are given as numbers of patients (percentage); Values for continuous variables are given as mean ± standard deviation. LA, left atrial; LV, left ventricular; TR, tricuspid regurgitation.

### Hyponatremia as a predictor of a new-onset cardiovascular event

**Fig 1:**
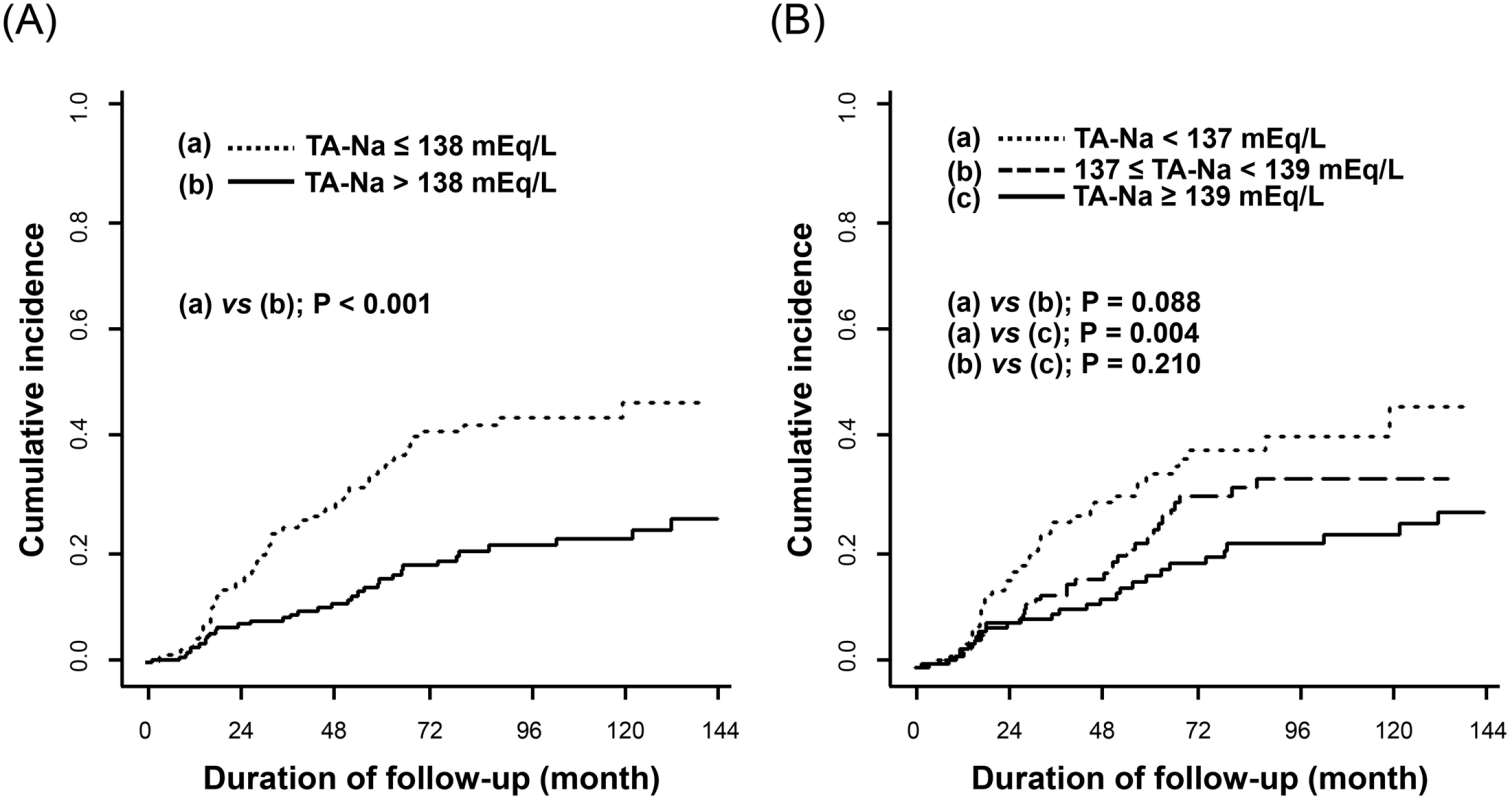
The cumulative incidence curves for the first occurrence of a cardiovascular event after the initiation of peritoneal dialysis between groups defined according to the median (A) and tertile (B) of the time-averaged serum sodium (TA-Na) levels.

During a mean follow-up time of 43.2 months, 106 (24.0%) patients developed new CV events including coronary artery disease (56 patients, 52.8%), cerebrovascular disease (26 patients, 24.5%), congestive heart failure (13 patients, 12.3%), and peripheral artery disease (11 patients, 10.4%). The cumulative incidence of new-onset CV events after initiation of PD was significantly higher in patients with lower TA-Na levels than in those with a TA-Na > 138 mEq/L (median) or a TA-Na ≥ 139 mEq/L (highest tertile), respectively (Fig 1).

A higher TA-Na was associated with a lower risk of a new CV event (Table 2). Models featuring various adjustments showed that this association was both significant and consistent. Using serum TA-Na as a continuous variable, the SHR for new-onset CV events was 0.90 per 1 mEq/L rise in TA-Na (95% CI, 0.83–0.96; *p* = 0.003), indicating that a higher TA-Na was significantly associated with a decreased risk of developing new CV events. In addition, patients with a TA-Na ≤ 138 mEq/L had a 2.31-fold higher risk of CV events than those with a TA-Na > 138 mEq/L. First-order interaction terms between covariates were determined for all models, but we found no evidence of any interaction between these covariates.

**Table 2:** Multivariable Cox regression analyses for the risk of new-onset cardiovascular events.

	Model 1			Model 2			Model 3		
	SHR	(95% CI)	*p*	SHR	(95% CI)	*p*	SHR	(95% CI)	*p*
**Continuous model**									
(per 1 mEq/L TA-Na increase)	0.89	(0.83–0.95)	<0.001	0.89	(0.83–0.95)	<0.001	0.90	(0.83–0.96)	0.003
**Categorical model**									
(Median)									
TA-Na ≤ 138 mEq/L	2.27	(1.65–3.68)	<0.001	2.57	(1.73–3.82)	<0.001	2.31	(1.49–3.58)	<0.001
TA-Na > 138 mEq/L	1.00	(reference)		1.00	(reference)		1.00	(reference)	
(Tertile)									
TA-Na < 137 mEq/L	2.07	(1.29–3.32)	0.003	2.22	(1.39–3.56)	0.001	1.94	(1.17–3.21)	0.010
137 mEq/L ≤ TA-Na <139 mEq/L	1.39	(0.86–2.26)	0.180	1.43	(0.89–2.32)	0.140	1.10	(0.65–1.88)	0.720
TA-Na ≥ 139 mEq/L	1.00	(reference)		1.00	(reference)		1.00	(reference)	

TA-Na, time-averaged serum sodium; SHR, subdistribution hazard ratio; CI, confidence interval. Adjustments in model 1: Demographic and clinical parameters including sex, body mass index, Charlson Comorbidity Index score, anti-hypertensive medications; model 2: model 1 plus dialysis-specific parameters including use of icodextrin, use of high glucose dialysate, peritoneal dialysis ultrafiltration, total Kt/V urea, normalized protein catabolic rate, and percentage of lean body mass; model 3: model 2 plus laboratory parameters including serum hemoglobin, serum albumin, calcium, phosphorus, potassium, bicarbonate, serum ferritin, C-reactive protein, and residual renal function.

To rule out that the results were influenced by the presence of previous CV disease, we repeated all competing risk analyses in subgroups of the 318 patients without previous CV disease. In this additional analysis, the observed effects in the various subgroups were similar to those in the crude analyses (data not shown). Finally, we also conducted subgroup analyses to evaluate the efficacy of serum sodium level as a predictor of cause-specific CV events. The unadjusted model revealed an increased risk of coronary artery disease (*p* = 0.017) in patients with TA-Na ≤ 138 mEq/L; however, the association was not significant after adjustment. Interestingly, all new-onset peripheral artery disease occurred in patients with TA-Na ≤ 138 mEq/L. In contrast, neither the unadjusted or adjusted analyses revealed any association between low serum sodium levels and the risks of congestive heart failure or cerebrovascular disease.

### Cumulative incidence estimates of the other outcomes

**Fig 2:**
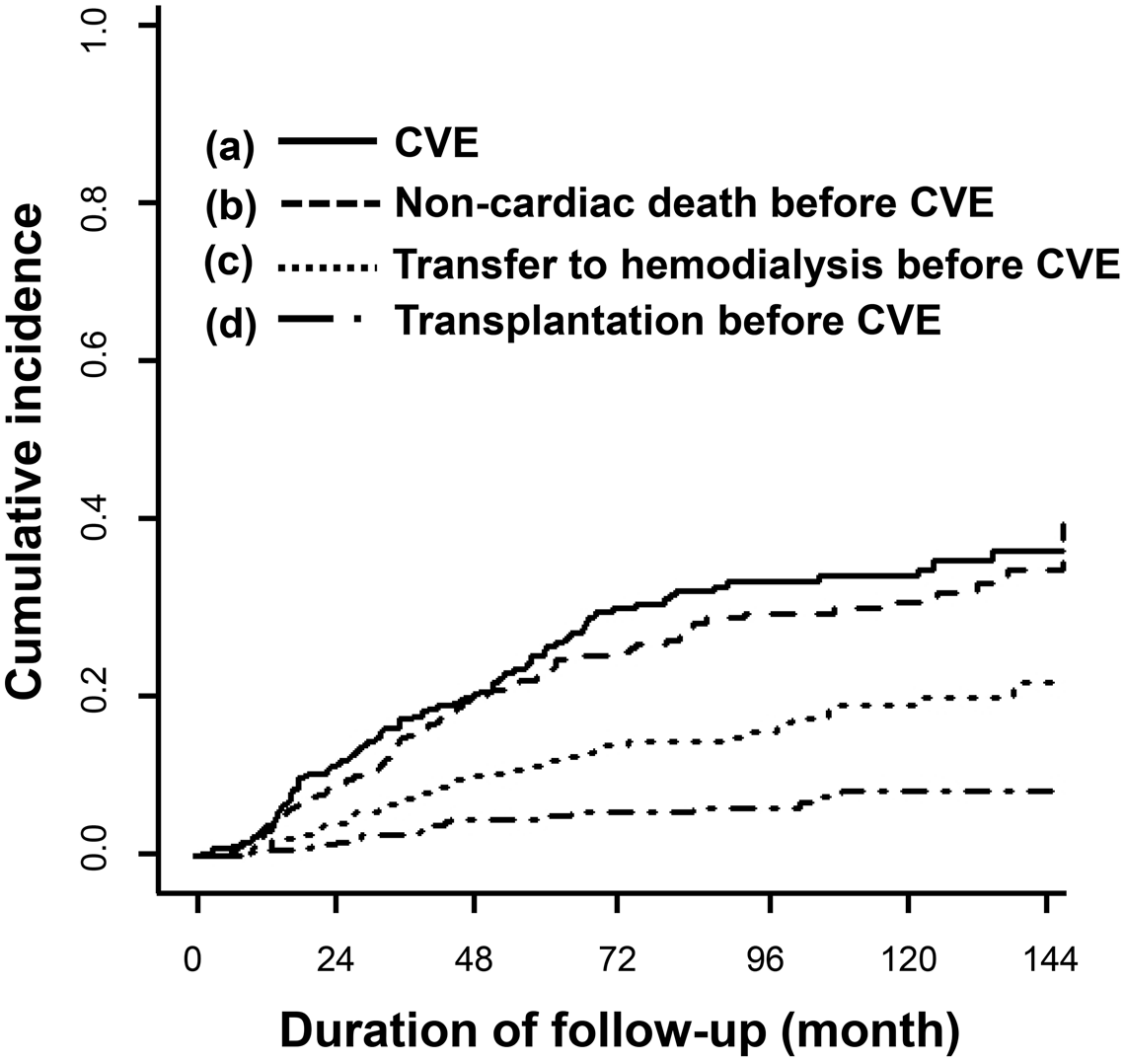
The cumulative incidence curves for a new-onset cardiovascular event and other outcomes. CVE, cardiovascular event.

The competing risk analysis also allows the calculation of the cumulative incidence of the other possible outcomes, shown in Fig 2 and summarized in Table 3. Non-CV death before any new-onset CV event was the second-rank outcome (cumulative incidence of 0.30 at 120 months), followed by transfer to HD (0.18), and renal transplantation (0.07) before a CV event (Fig 2).

**Table 3:** Cumulative incidence of a new-onset cardiovascular event and other outcomes.

Duration of follow up (months)	24	48	72	96	120
**(Median)**					
CVE					
TA-Na ≤ 138 mEq/L	0.14	0.28	0.40	0.43	0.45
TA-Na > 138 mEq/L	0.07	0.10	0.18	0.21	0.23
Non-cardiovascular death before CVE					
TA-Na ≤ 138 mEq/L	0.09	0.19	0.22	0.24	0.27
TA-Na > 138 mEq/L	0.07	0.19	0.27	0.31	0.34
Transfer to hemodialysis before CVE					
TA-Na ≤ 138 mEq/L	0.06	0.10	0.15	0.15	0.17
TA-Na > 138 mEq/L	0.02	0.09	0.11	0.15	0.19
Transplantation before CVE					
TA-Na ≤ 138 mEq/L	0.00	0.03	0.04	0.04	0.06
TA-Na > 138 mEq/L	0.03	0.05	0.06	0.07	0.09
**(Tertile)**					
CVE					
TA-Na < 137 mEq/L	0.16	0.29	0.38	0.41	0.46
137 mEq/L ≤ TA-Na <139 mEq/L	0.08	0.17	0.30	0.33	0.33
TA-Na ≥ 139 mEq/L	0.08	0.12	0.19	0.22	0.25
Non-cardiovascular death before CVE					
TA-Na < 137 mEq/L	0.11	0.21	0.25	0.25	0.25
137 mEq/L ≤ TA-Na <139 mEq/L	0.07	0.16	0.20	0.24	0.27
TA-Na ≥ 139 mEq/L	0.07	0.18	0.27	0.32	0.36
Transfer to hemodialysis before CVE					
TA-Na < 137 mEq/L	0.06	0.11	0.15	0.15	0.15
137 mEq/L ≤ TA-Na <139 mEq/L	0.04	0.10	0.13	0.15	0.19
TA-Na ≥ 139 mEq/L	0.02	0.08	0.11	0.15	0.19
Transplantation before CVE					
TA-Na < 137 mEq/L	0.00	0.05	0.05	0.05	0.08
137 mEq/L ≤ TA-Na <139 mEq/L	0.01	0.03	0.04	0.04	0.04
TA-Na ≥ 139 mEq/L	0.04	0.05	0.06	0.08	0.11

CVE, cardiovascular event;

TA-Na, time-averaged serum sodium.

## Discussion

In this study, we investigated the relationship between serum sodium levels and the occurrence of new-onset CV events in our cohort of patients with ESRD undergoing PD. We showed that a low TA-Na level independently predicted new-onset CV events, suggesting that hyponatremia is a potential predictor of adverse outcomes in PD patients. Thus, patients with low sodium levels should be receive appropriate treatment to prevent CV disease even after initiation of dialysis.

Hyponatremia has long been described as a predictor of CV mortality and morbidity in severely ill patients, especially in those with heart failure, myocardial infarction, stroke, or cirrhosis [3, 4, 15–17]. Several recent studies, including ours, have shown that hyponatremia is common among patients with ESRD and is strongly associated with poor clinical outcomes [6–10]. However, these studies focused primarily on mortality risk. Patients with ESRD who are on maintenance dialysis are at an increased risk of mortality; moreover, CV disease is the leading cause of hospitalization and death in patients on PD [11]. Nonetheless, few studies have investigated the association between serum sodium levels and the risk of developing CV disease in patients on PD.

Our results indicate that a low serum sodium concentration is significantly associated with an increased risk of new CV events in patients undergoing PD. Even after vigorous adjustments for potentially multiple confounding covariates, including demographic, laboratory, and dialysis-specific data, an increase in the TA-Na level of 1 mEq/L was still associated with a 10% decreased risk of newly developing a CV event. Moreover, patients with a TA-Na level ≤ 138 mEq/L had a 2.31-fold higher risk of CV events than patients with a TA-Na level > 138 mEq/L, indicating the significant association between higher serum sodium levels and a lower risk of a newly developed CV event.

The mechanisms underlying the increased risk of CV events in PD patients with low serum sodium levels are complex and poorly understood. Previous studies have focused on patients with chronic heart failure [18–22], in which hyponatremia was shown to be related to neurohormonal activation, such as the non-osmotic release of vasopressin [20], activation of the renin-angiotensin system [21], and increased catecholamine production [22]. However, any relationship between neurohormonal activation and adverse outcomes in patients undergoing PD is largely unknown [23]; thus, it is unclear whether mechanisms contributing to the development of chronic hyponatremia in patients with normal GFRs can explain our findings. Future studies are needed to evaluate the effect of neurohormonal activation on the development of hyponatremia and CV disease in PD patients. Moreover, several factors associated with the development of hyponatremia in patients with ESRD, such as reduction in residual renal function, protein-energy wasting, and chronic inflammation are likely to underlie the high risk of CV events in patients undergoing PD [24–27]. Furthermore, these factors are well-established predictors of mortality and have been shown to play pivotal roles in the development of CV disease in patients on dialysis [28–31]. Taken together, the above mechanisms may explain the association between hyponatremia and the increased CV risk in patients undergoing PD.

Our study had several limitations. As an observational study, the causality of our findings needs further confirmation. Furthermore, it is unknown whether the correction of hyponatremia can improve clinical outcomes in dialysis patients. Moreover, because of our relatively small sample size, we were not able to determine conclusively the effect of hyponatremia on cause-specific CV disease. Finally, given that the mechanism underlying hyponatremia is likely multifactorial [24], additional information, on nutritional status and neurohormonal activation, is needed to explain the development of hyponatremia in patients with CKD. In particular, lack of data regarding volume status is important drawback, despite baseline echocardiographic parameters related to the state of hydration were partly presented in 98 patients; a more judicious approach using the objective techniques such as echocardiographic parameters, serum biomarkers, and bioelectrical impedance analysis might be warranted to assess fluid status in hyponatremic PD patients. Nonetheless, this study showed that a low TA-Na level independently predicted the development of new-onset CV events after dialysis initiation, even after extensive adjustment for demographic, clinical, laboratory, and dialysis-specific covariates. Our robust findings suggest that hyponatremia portends a poor prognosis; thus, in clinical practice, physicians should be meticulous in treating both the electrolyte imbalance and the underlying disease.

### Conclusion

In conclusion, our study showed that a low serum sodium concentration is an independent predictor of new-onset CV events in PD patients. Whether the correction of hyponatremia in this setting provides additional protection against the development of CV events will need to be addressed in interventional studies.
